# Phytocytokines function as immunological modulators of plant immunity

**DOI:** 10.1007/s44154-021-00009-y

**Published:** 2021-09-15

**Authors:** Shuguo Hou, Derui Liu, Ping He

**Affiliations:** 1 School of Municipal & Environmental Engineering, Shandong Jianzhu University, Jinan, 250100 China; 2 Department of Biochemistry & Biophysics, Texas A&M University, College Station, TX 77843 USA

**Keywords:** Phytocytokine, Damage-associated molecular pattern (DAMP), Pattern-recognition receptor (PRR), pattern-triggered immunity (PTI), Plant immunity

## Abstract

Plant plasma membrane-resident immune receptors regulate plant immunity by recognizing microbe-associated molecular patterns (MAMPs), damage-associated molecular patterns (DAMPs), and phytocytokines. Phytocytokines are plant endogenous peptides, which are usually produced in the cytosol and released into the apoplast when plant encounters pathogen infections. Phytocytokines regulate plant immunity through activating an overlapping signaling pathway with MAMPs/DAMPs with some unique features. Here, we highlight the current understanding of phytocytokine production, perception and functions in plant immunity, and discuss how plants and pathogens manipulate phytocytokine signaling for their own benefits during the plant-pathogen warfare.

## Introduction

In the early 1990s, systemin was identified as the first peptide signaling molecule in tomato (Pearce et al. 1991). Over the past three decades, dozens of small peptides belonging to different families have been identified and functionally characterized as signaling molecules in various plant species, especially in reference plants *Arabidopsis thaliana*. Substantial evidence indicates that these peptides, similar to conventional phytohormones, are highly active at a low concentration and play critical roles in the regulation of plant development, reproduction, immunity, and adaptation to environmental stresses. However, different from classic phytohormones which are biosynthesized through specialized metabolic reactions and have conserved structures and functions across the plant kingdom, these peptides are gene products with high sequence diversity and functional specificity across plant species (Matsubayashi 2014; Olsson et al. 2019; Takahashi et al. 2019).

In recent years, different types of small secreted peptides have been implicated in regulating plant immunity. Genes encoding many of these immunomodulatory peptides are rapidly and substantially induced during pathogen infections or treatments with pathogen/microbe-associated molecular patterns (PAMPs/MAMPs) (Li et al. 2016a). Recognition of MAMPs by plant plasma membrane (PM)-resident pattern recognition receptors (PRRs) initiates plant pattern-triggered immunity (PTI), the first line of inducible defense against infections (Couto and Zipfel 2016; Yu et al. 2017; Zhou and Zhang 2020). Plant PRRs also recognize host-derived damage-associated molecular patterns (DAMPs), such as extracellular nucleotides, fragments of plant cell wall-derived polysaccharides and immune-related proteins and peptides, which are usually released upon cell damages (Gust et al. 2017; Hou et al. 2019a; Tanaka and Heil 2021). Although immunomodulatory peptides were regarded as a type of DAMPs, most immunological peptides are secreted into extracellular apoplasts in the absence or before cell damages. Furthermore, immunological peptides have different chemical characteristics, maturation processes, and mode-of-actions compared to the conventional DAMPs. These immunological peptides are functionally analogous to animal cytokines, a group of signaling peptides produced by immune cells, endothelial cells, and fibroblasts, functioning in health and disease, especially in host immunity, inflammation, trauma, sepsis, and cancer as immunomodulating agents (Banchereau et al. 2012; Luo 2012). Therefore, plant cytokines or phytocytokines were coined to refer to plant peptide hormones that regulate both plant immunity and development as signals of cell-cell communication (Luo 2012). Hence, plant-derived immunogenic factors were further divided into two categories with one class as classical DAMPs: molecules that are passively released upon cell damage, and one class as phytocytokines: immunomodulatory peptides that are processed and/or secreted upon infections (Gust et al. 2017; Tanaka and Heil 2021).

Notably, some peptide hormones initially identified as regulators of plant development, reproduction or abiotic stress response have been shown to be involved in plant immunity. Similarly, some immunological peptides also play roles in other physiological processes. Thus, those immunological phytocytokines have dual roles in plant immunity, development, growth, reproduction or stress adaptation, similar to cytokines in animal physiology. Interestingly, some phytocytokine-like sequences were identified in microbes, which could activate or attenuate plant immunity. In this review, we will highlight the recent advances on the mechanisms of phytocytokine-mediated plant immunity and discuss how plants and phytopathogens manipulate phytocytokine signaling for the survival of each site during the host-pathogen interactions.

## Classification, identification and function of phytocytokines

The phytocytokines can be divided into two major classes based on whether their precursor proteins contain a signal peptide (Matsubayashi 2014). Systemin, plant elicitor peptide (PEP1), *Z. mays* immune signaling peptide 1 (ZIP1), and soybean *Gm*PEPs belong to the group of phytocytokines whose precursors are absent of a signal peptide and are classified as non-secreted peptides. Other phytocytokines, including hydroxyproline-rich systemins (HypSys), PAMP-induced secreted peptide 1 (PIP1)/PIP2, serine-rich endogenous peptide 12 (SCOOP12), phytosulfokines (PSKs), plant peptide containing sulphated tyrosine 1 (PSY1), inflorescence deficient in abscission (IDA)/IDA-LIKE 6 (IDL6), root meristem growth factors (RGFs)/GOLVENs (GLVs), and rapid alkalization factors (RALFs) constitute the other group whose precursors harbor a signal peptide and are classified as secreted peptides (Table 1).

**Table 1:** Classification, perception and functions of phytocytokines

Type	Phytocytokines	Receptor	Functions	Reference
Non-secreted peptides	Systemin	SYR1	Induction of proteinase inhibitors, extracellular alkalization, and ethylene emission, mediation of systemic defense response, defense against insect herbivory	Pearce et al. 1991; Ryan and Pearce 2003; Wang et al. 2018
	Pep1, Pep2, Pep3	PEPR1, PEPR2	Activation of PTI responses, and plant resistance to *B. cinerea*, *P. syringae*, and *P. irregular*, activation of ET, JA, and SA signaling pathways, mediation of systemic immunity	Huffaker et al. 2006, 2011; Liu et al. 2013; Ross et al. 2014; Yamaguchi et al. 2006, 2010
	ZIP1	Unknown	Activation of SA defense signaling, maize resistance against *U. maydis* and susceptibility to *B. cinerea*	Ziemann et al. 2018
	GmPep914, GmPep890	Unknown	Induction of extracellular alkalization and the expression of defense genes	Yamaguchi et al. 2011
Secreted peptides	PSK	PSKR1	Attenuation of PTI and SA signaling, activation of JA signaling, increase resistance to necrotrophic pathogens and susceptibility to biotrophic pathogens	Amano et al. 2007; Igarashi et al. 2012; Matsubayashi et al. 2002; Matsubayashi and Sakagami 1996; Mosher et al. 2013; Rodiuc et al. 2016; Zhang et al. 2018
	PSY1	PSY1R?		
	PIP1, PIP2	RLK7	Activation of PTI responses and plant resistance to *P. syringae* and *F. oxysporum*	Hou et al. 2014
	IDA	HAE, HSL2	Regulation of plant resistance to *P. syringae*	Butenko et al. 2003; Patharkar and Walker 2016; Stenvik et al. 2008; Wang et al. 2017
	IDL6	HAE, HSL2	Suppression of PTI responses and SA signaling, attenuation plant resistance to *P. syringae*	
	SCOOP12	MIK2	Activation of PTI responses, plant resistance to *P. syringae* and *F. oxysporum*, and susceptible to *E. amylovora*	Gully et al. 2019; Hou et al. 2021a, b; Rhodes et al. 2021
	RGF7/GLV4	RGI3/4	Activation of PTI responses	Matsuzaki et al. 2010; Ou et al. 2016; Stegmann et al. 2021; Wang et al. 2021; Whitford et al. 2012
	RGF9/GLV2	RGI3	Activation of PTI responses Promotion of FLS2 accumulation	
	HypSys	Unknown	Induction of proteinase inhibitors, extracellular alkalization, and ethylene emission, activation of resistance to insect herbivory	Pearce et al. 2001a, b; Pearce and Ryan, 2003
	RALF1	FER-LLG	Attenuation of PTI, activation of JA signaling	Haruta et al. 2014; Li et al. 2015; Stegmann et al. 2017; Guo et al. 2018
	RALF17	Unknown	Activation of PTI responses	
	RALF22, RALF23	FER-LLG	Attenuation of PTI signaling	

Systemin is an 18-amino acid (aa) peptide, identified from wounded tomato leaf extracts and named as it can mediate long-distance systemic defense responses (McGurl et al. 1992; Pearce et al. 1991). Systemin was found in most species of the Solanaceae family (Ryan and Pearce 2003). Treatment of tomatoes with systemin triggers an array of resistance responses, including the production of proteinase inhibitors, the induction of extracellular alkalization and ethylene emission, and defense against insect herbivory (Zhang et al. 2020a). PEP1, a 23-aa peptide, is the first phytocytokine identified in Arabidopsis. Arabidopsis genome encodes eight PEPs, and their orthologs have been identified in a wide array of plant species, including maize, rice, potato, and soybean (Huffaker et al. 2011; Huffaker et al. 2006; Poretsky et al. 2020). *At*PEP1 activates the hallmark of PTI responses, promotes plant resistance to various pathogens, including bacterium *Pseudomonas syringae*, fungus *Botrytis cinerea* and oomycete *Phytophthora infestans* (Huffaker et al. 2006; Yamaguchi et al. 2010; Liu et al. 2013). ZIP1 is a 17-aa peptide isolated from apoplastic fluids of salicylic acid (SA)-pretreated leaves in maize. ZIP1 treatment strongly elicits SA accumulation, induces highly overlapping transcriptional changes associated with SA-responsive genes, and increases maize resistance against the biotrophic fungus *Ustilago maydis* but susceptibility toward the necrotrophic fungus *B. cinerea* (Ziemann et al. 2018). *Gm*PEP914 and *Gm*PEP890 are two homologous eight-aa peptides isolated from leaf extracts of soybean and identified as alkalization factors of suspension-cultured cells (Yamaguchi et al. 2011). Both peptides can induce the expression of defense genes involved in pathogen defense.

HypSys are a group of homologous hydroxyproline-rich glycopeptides identified in Solanaceae and Convolvulaceae family plants (Chen et al. 2008; Narvaez-Vasquez et al. 2005; Pearce et al. 2001a). HypSys have similar sizes and functions with those of systemin but do not share sequence homology with systemin. PIP1 and PIP2 peptides are corresponding to the C-termini of two secreted peptide precursor proteins, prePIP1 and prePIP2, identified as MAMP-induced gene products (Hou et al. 2014). Arabidopsis harbors 11 prePIP paralogs, and prePIP orthologs have been found in a large number of monocot and eudicot species. PIP1 and PIP2 are able to activate classical PTI responses and enhance Arabidopsis resistance to *P. syringae* pv. *tomato* (*Pst*) DC3000 and *Fusarium oxysporum* (Hou et al. 2014). Like PIP1 and PIP2, SCOOP12 is derived from the C-terminus of a pathogen-responsive secreted peptide precursor, PROSCOOP12 (Gully et al. 2019). At least 23 PROSCOOPs have been identified in Arabidopsis (Hou et al. 2021a; Rhodes et al. 2021). PROSCOOP orthologs are only found in Brassicaceae family plants. Most SCOOP peptides trigger various PTI responses or/and resistance to *Pst* DC3000 (Gully et al. 2019; Hou et al. 2021a; Rhodes et al. 2021; Yu et al. 2020). Arabidopsis plants defective in *SCOOP12* are more resistant to *Erwinia amylovora* (Gully et al. 2019). Similar to HypSys, PIPs and SCOOPs, IDA is a serine and glycine-rich peptide. It was initially identified as a key regulator of floral organ abscission in Arabidopsis (Butenko et al. 2003). IDA homologs are conserved in a wide range of plant species (Butenko et al. 2003). The IDA family comprises eight members in Arabidopsis (Vie et al. 2015). Of these, IDA and IDL6 have been reported to be involved in plant immunity. IDA regulates plant resistance likely through the control of premature leaf abscission (Patharkar and Walker, 2016). IDL6 promotes Arabidopsis susceptibility to Pst DC3000 (Wang et al. 2017).

PSKs are five-aa peptides with two sulfated tyrosine residues. They were initially identified as a plant growth-promoting factor and have been found to regulate multiple processes of plant growth, development, and stress responses (Matsubayashi and Sakagami 1996; Sauter 2015; Yang et al. 2001). PSKs are universally distributed in the plant kingdom. In Arabidopsis, PSK signaling attenuates PTI responses, compromises plant resistance to the hemibiotrophic *Pst* DC3000 and the oomycete *Hyaloperonospora arabidopsidis*, but enhances resistance against the necrotrophic fungal pathogen *Alternaria brassicicola* (Igarashi et al. 2012; Mosher et al. 2013; Rodiuc et al. 2016). In tomato, PSK enhances the resistance to necrotrophic fungal pathogen *B. cinerea* (Zhang et al. 2018). Arabidopsis PSY1 is an 18-aa glycopeptide with a sulfated tyrosine residue. It was originally identified as a functional analogy to PSKs in stimulating plant cellular proliferation and expansion (Amano et al. 2007). PSY homologs have been identified in diverse plant species, including rice, banana, tomato, and wheat (Pruitt et al. 2017). Like PSKs, PSY1 signaling likely suppresses PTI responses, promotes Arabidopsis resistance to *A. brassicicola* but susceptibility to *Pst* DC3000 and *F*. *oxysporum* (Mosher et al. 2013; Shen and Diener 2013). RGF family peptides also known as GLV peptides represent another group of tyrosine-sulfated peptides that were initially identified as key regulators of root meristem maintenance and gravitropism in Arabidopsis (Matsuzaki et al. 2010; Whitford et al. 2012). The RGF peptide family comprises 11 members in Arabidopsis (Matsuzaki et al. 2010). Among them, RGF7/GLV4 and RGF9/GLV2, which are transcriptionally regulated in plants upon infection by *P. syringae*, contribute to the activation of immune responses and the increase of resistance to *P. syringae* (Stegmann et al. 2021; Wang et al. 2021).

RALFs, a group of 5-kilodalton (kDa) polypeptides originally extracted from tobacco leaves, induces rapid alkalization of extracellular compartment and functions in root growth and development (Pearce et al. 2001b). Different from other linear peptides, RALFs have four conserved cysteines which form two disulfide bonds that are vital to the peptide activity. RALFs are widely present in various tissues and organs of different plant species (Pearce et al. 2001b). Arabidopsis genome encodes more than 30 RALFs, and some of them have been shown to play a positive or negative role in plant immunity (Blackburn et al. 2020) (Table 1).

## Phytocytokines are perceived by cell surface receptors

A major common feature of cytokines and phytocytokines is that they are perceived by specific cell surface receptors. Receptors for cytokines are structurally diverse and mainly divided into five major superfamilies: type I (hematopoietin family) and type II (interferon family) cytokine receptors, tumor necrosis factor (TNF) family receptors, immunoglobulin superfamily receptors, receptor tyrosine kinases, and chemokine receptors (Wang et al. 2009). In contrast, phytocytokines are usually perceived by cell surface-resident receptor-like kinases (RLKs), which contain an extracellular domain, a transmembrane region, and a cytoplasmic kinase domain resembling the animal receptor tyrosine kinases (Couto and Zipfel 2016; Escocard de Azevedo Manhaes et al. 2021; Shiu and Bleecker 2001) (Table 1). Plant RLKs are classified into different subfamilies based on their extracellular domains. Leucine-rich repeat-RLKs (LRR-RLKs) with extracellular LRRs constitute the largest subfamily of RLKs and function as receptors of some immunological phytocytokines. Of these, tomato SYSTEMIN RECEPTOR 1 (SYR1) and SYR2 perceive systemin (Wang et al. 2018), Arabidopsis PEP1 RECEPTOR 1 (PEPR1)/PEPR2 recognize PEPs (Yamaguchi et al. 2006), Arabidopsis RECEPTOR-LIKE 7 (RLK7) recognizes PIP1 and PIP2 (Hou et al. 2014), Arabidopsis MALE DISCOVERER 1-INTERACTING RECEPTOR-LIKE KINASE 2 (MIK2) recognizes SCOOPs (Hou et al. 2021a; Rhodes et al. 2021), HAESA and HAESA-LIKE2 (HSL2) recognize IDA (Santiago et al. 2016), RGF1 INSENSITIVE 3 (RGI3) itself or together with RGI4 recognizes RGF7 and RGF9/GLV2 (Stegmann et al. 2021; Wang et al. 2021), PSK RECEPTOR 1 (PSKR1) recognizes PSKs (Matsubayashi et al. 2002; Wang et al. 2015), and PSY1R likely recognizes PSY1 (Amano et al. 2007). These phytocytokine receptors all belong to LRR X and LRR XI clades of LRR-RLKs, which are phylogenetically close to the LRR XII subfamily of LRR-RLKs, including some of the well-studied receptors of proteinaceous MAMPs, such as the bacterial flagellin receptor FLAGELLIN SENSING 2 (FLS2) and ELONGATION FACTOR-Tu RECEPTOR (EFR). This suggests a close evolutionary relationship between the phytocytokine- and MAMP-triggered immunity. Upon phytocytokine perception, the LRR-RLK receptors often heterodimerize with SOMATIC EMBRYOGENESIS RECEPTOR-LIKE KINASE (SERK) LRR-RLKs, e.g., BRASSINOSTEROID INSENSITIVE 1 (BRI1)-ASSOCIATED RECEPTOR KINASE 1 (BAK1)/SERK3 and SERK4 (Liu et al. 2020b; Ma et al. 2016).

*Catharanthus roseus* receptor-like kinase 1-like (CrRLK1L) proteins with two extracellular malectin-like domains play important roles in plant development, such as polarized growth, cell elongation, cell wall integrity sensing, and hormonal responses (Franck et al. 2018; Li et al. 2016b; Zhu et al. 2021). Recent studies suggest a critical role of CrRLK1Ls in plant immunity as receptors of RALFs. Arabidopsis CrRLK1L FERONIA (FER) functions as the receptor of different RALFs, including RALF1 and RALF23 (Haruta et al. 2014; Stegmann et al. 2017) (Table 1). Interestingly, RALF23/RALF33 negatively, whereas RALF17 positively regulates PRR-mediated immunity in a FER-dependent manner (Stegmann et al. 2017). Structural and biochemical results indicate that RALF23 induces a complex formation between FER and LORELEI (LRE)-LIKE GLYCOSYLPHOSPHATIDYLINOSITOL (GPI)-ANCHORED PROTEIN 1 (LLG1) or LLG2 to assemble a RALF23-LLG1/2-FER ternary complex (Xiao et al. 2019). Although LLGs were initially proposed as coreceptors of FER, they could directly bind to RALFs without FER. It appears that RALF23 is initially recognized by LLGs, resulting in the recruitment of FER to the heteromeric complex (Xiao et al. 2019). Thus, LLGs might be the bona fide RALF receptors whereas CrRLK1Ls act as the coreceptors to strengthen the interaction.

## Regulation of phytocytokine expression

Regulating the expression of phytocytokine precursors is one of the early immune responses (Li et al. 2016a). Accordingly, some phytocytokines are identified as they are upregulated by MAMP treatments or pathogen infections. For example, *prePIP1* and *prePIP2*, the precursors of PIP1 and PIP2, were identified through the analysis of MAMP-regulated gene transcription (Hou et al. 2014). The expression of *prePIP1* and *prePIP2* is swiftly upregulated 30 min post-treatment with bacterial MAMP flg22 or elf18 and reaches a peak about one-hour post-treatment. Likewise, the expression of *prePIP1* is also highly induced by chitin, a MAMP from fungi, suggesting that prePIP1 may play a conserved role in plant resistance to diverse pathogens. Consistently, the expression of *prePIP1*in leaves and in roots is induced by the bacterial *Pst* DC3000 and the fungal *F. oxysporum* f. sp. *conglutinans* strain 699 (*Foc 699*), respectively, and PIP1 promotes plant resistance to both *Pst* DC3000 and *Foc 699* (Hou et al. 2014). *PROSCOOP12* was identified as it is highly induced by infections of diverse pathogens, including *B. cinerea*, *Pst* DC3000, and *E. amylovora* (Gully et al. 2019); *preRGF7* was induced by *Pst* DC3000 infection at transcriptional and post-transcriptional levels (Wang et al. 2021). Two *PROPEP1* paralogs, *PROPEP2* and *PROPEP3* are highly induced by MAMPs or pathogens, including *Pst* DC3000, *B. cinerea*, and *P. infestans* (Huffaker et al. 2006); the precursors of PSK and PSY1, *proPSKs* and *proPSY1*, were upregulated during the infection of *B. cinerea* or *A. brassicicola* in *Arabidopsis* and tomato (Igarashi et al. 2012; Mosher et al. 2013; Zhang et al. 2018). Consistent with the up-regulation of phytocytokine genes, the expression of their receptors is also upregulated by MAMPs in some cases. For instance, MAMP treatment or pathogen infection induce the expression of *PEPR1*, *PEPR2*, *RLK7*, *MIK2*, *HAESA*, and *PSKR1* (Kemmerling et al. 2011; Lewis et al. 2015). Interestingly, some phytocytokine precursor genes are transcriptionally downregulated by pathogen infections. For instance, the expression of *preRGF9*/*GLV2* is suppressed in leaves upon infection with *P. syringae* pv. *maculicola* and *Pst* DC3000 (Stegmann et al. 2021). It remains to be determined how down-regulation of *preRGF9*/*GLV2* plays a positive role in plant immunity.

MAMP-induced phytocytokine genes may further up-scale the expression of their precursor genes, thus amplifying the phytocytokine signaling through a positive feedback loop. For example, Pep1, PIP1, SCOOP12, and RGF7 are able to induce the expression of their precursor genes, respectively (Gully et al. 2019; Hou et al. 2014; Huffaker et al. 2006; Wang et al. 2021). Inducible overexpression of *preRGF7* in plants leads to the activation of MPK3 and MPK6, and the activated MPK3 and MPK6 in turn upregulate *preRGF7* expression via the downstream WRKY33 transcription factor, suggesting a self-amplification loop in the regulation of *preRGF7* expression (Wang et al. 2021). The discovery of MIK2 as the SCOOP receptor is enlightened by this positive feedback regulation. Activation of MIK2 kinase in a RLK7-MIK2 chimeric receptor (extracellular domain of RLK7 fused with transmembrane and intracellular domains of MIK2) upon PIP1 treatment induced the expression of some *PROSCOOP* genes, which were consequently confirmed to be the ligands of MIK2 (Hou et al. 2021a). In addition, some phytocytokines may not only induce the expression of their own precursor genes but also precursor genes of other phytocytokines. For instance, PIP1 and Pep1 can upregulate the expression of each other’s precursor genes (Hou et al. 2014), indicating a signaling network regulated by different phytocytokines.

## Regulation of phytocytokine maturation and release

**Fig. 1:**
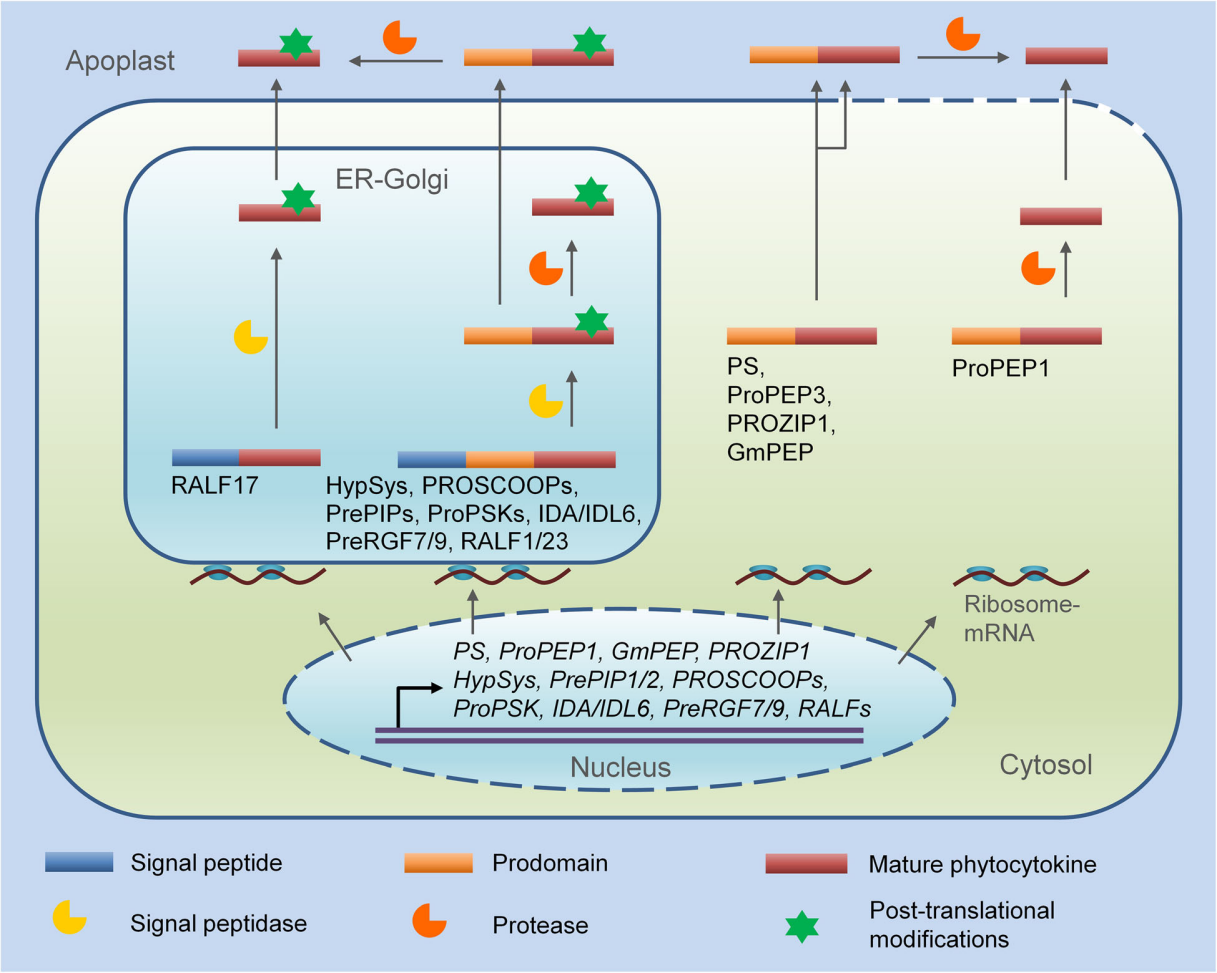
A model of phytocytokine maturation and release. After translation, phytocytokine precursors with signal peptide enter into the ER-Golgi secretory pathway, where they undergo post-translational modifications and proteolytic cleavages to remove signal peptide and prodomain, and then are secreted into the apoplast. Phytocytokine precursors without signal peptide enter into the cytosol, where they are maturely processed to remove prodomains. This group of phytocytokines are released into the apoplast likely through unconventional secretion or disrupted plasma membrane. The maturation processing of phytocytokine may also happen in the apoplast

As mentioned above, phytocytokines are usually derived from precursor proteins with the following characteristics: an amino (N)-terminal signal peptide (only for secreted peptides), a carboxyl (C)-terminal region conserved in the same family of peptides, and a variable region (also named prodomain) between signal peptide and conserved region (Fig. 1) (Matsubayashi 2014; Olsson et al. 2019). Once translated, the phytocytokine precursors enter the secretory pathway with the guide of signal peptide and are finally secreted into the extracellular compartment (apoplast) of plant cells as biologically active, mature phytocytokines. In the secretory pathway of endoplasmic reticulum (ER) and Golgi or the apoplast, proteolytic cleavages of signal peptide and prodomain, and post-translational modifications, such as tyrosine sulfation, proline hydroxylation, hydroxyproline arabinosylation, and intramolecular disulfide bond formation, are required for the phytocytokine maturation (Matsubayashi 2014; Olsson et al. 2019). For the phytocytokine precursors without a signal peptide (non-secreted peptides), they do not enter the canonical ER-Golgi secretory pathway and undergo post-translational modifications and are proposed to be released into the extracellular compartment via an unconventional secretory pathway or during cellular damage (Ding et al. 2012). The processing of the phytocytokine precursors to remove their prodomains that happens in the cytosol or the apoplast is also essential for their maturation (Fig. 1).

Substantial studies demonstrate that the maturation and secretion/release of phytocytokines are promoted by pathogen infections or other environmental stresses (Hou et al. 2021b). PROPEP1, the precursor protein of PEP1, lacks a canonical N-terminal signal sequence and is tethered on the cytosolic side of the tonoplasts. Arabidopsis METACASPASE 4 (MC4) and the other type-II metacaspases (MC5 to MC9) are required for PEP1 cleavage from PROPEP1 after an arginine residue (R69) (Hander et al. 2019; Shen et al. 2019). Plant metacaspases are structurally homologous to animal caspases (Hou et al. 2018). Arabidopsis MC4 is activated when cytosolic Ca^2+^ concentration reaches a threshold during cell membrane disruption or the MAMP flagellin peptide flg22-triggered Ca^2+^ elevation in cytosol (Hander et al. 2019; Shen et al. 2019). Recent structural studies revealed the molecular basis of Ca^2+^-dependent MC4 activation and PROPEP1 processing (Zhu et al. 2020). In addition, infection with either virulent or avirulent bacterial pathogens, salt stress treatment, and cell wall damage (CWD) also induce PROPEP3 cleavage to PEP3 (Engelsdorf et al. 2018; Yamada et al. 2016). It is unclear whether the elevation of cytosolic Ca^2+^ and MC activation are responsible for the induced PROPEP3 cleavage. Cell membrane damage might be a cause of PEP release, but it’s puzzling how PEP is released during PTI and salt stress when no observable cell membrane disruption is involved.

Tomato systemin is processed from the C-terminus of a precursor protein, prosystemin (PS) by two tomato subtilases, SLPHYTASPASE 1 (*Sl*Phyt1) and *Sl*Phyt2, at two aspartate residues (Beloshistov et al. 2018). Subtilases are a family of subtilisin-like serine proteases (Hou et al. 2018). *Sl*Phyt-mediated cleavage produces a modified systemin with an extra leucine residue at the N-terminus (Leu-systemin), which is further processed by a leucine aminopeptidase to generate mature systemin (Beloshistov et al. 2018). PSKs are also maturely processed from their precursor proPSKs after an aspartate residue by *Sl*Phyt2 in tomato. The PSK processing in tomato abscission region is induced by drought stress (Reichardt et al. 2020). However, proPSK1 and proPSK4 are processed by subtilases SBT1.1 and SBT3.8 respectively in Arabidopsis (Srivastava et al. 2008; Stuhrwohldt et al. 2021). The cleavage of IDA precursor proteins is executed by SBT4.12, SBT4.13, and SBT5.2 (Schardon et al. 2016).

Some RALFs, including RALF1, RALF22, and RALF23, contain a signal peptide and a RRXL cleavage site in the junction region between prodomain and the mature RALF peptides, which is cleaved by the ER-localized subtilase SITE-1 PROTEASE (S1P) (Srivastava et al. 2009; Stegmann et al. 2017). Treatment with elf18, an epitope peptide of MAMP elongation factor Tu (EF-Tu), or inoculation with *Pst* DC3000 *hrcC* mutant, which is deficient in the bacterial type III secretion system, significantly promotes the processing of the PRORALF23 (Stegmann et al. 2017). This cleavage leads to the activation and secretion of RALF peptides as active phytocytokines. The RALFs without the RRXL cleavage site, such as RALF17, are not cleaved by S1P (Stegmann et al. 2017). ZIP1 is processed from PROZIP1 by maize papain-like cysteine protease (PLCP) CP1 and CP2 (Ziemann et al. 2018). The processing of ZIP1 is promoted by SA, an immune-related phytohormone that is usually highly induced in plants upon attacks of biotrophic and hemibiotrophic pathogens (Ziemann et al. 2018). ZIP1 strongly elicits SA accumulation and activates PLCP in maize leaves, indicating a positive feedback loop in regulating ZIP1 signaling (Ziemann et al. 2018). Like PROPEP1, both PS and PROZIP1 lack canonical signal peptides. Notably, SlPhyts and CPs are apoplastic proteases. Therefore, it is possible that PS and PROZIP1 are released into extracellular spaces during cellular damages or through unconventional protein secretory pathways and then processed to mature systemin and ZIP1 in apoplasts. Other phytocytokines, including HypSys, PIP1, PIP2, and SCOOP12, were also shown to be processed (Hou et al. 2021a; Hou et al. 2014; Pearce et al. 2001a), but the proteases that mediate the cleavage have not been identified yet. Precursors of HypSys, IDA, PIP1, PIP2, and SCOOP12 contain typical signal peptide, and are supposed to be secreted into apoplasts. It remains unknown whether the cleavage of these precursors occurs in the cytoplasm or apoplasts to become the mature phytocytokines.

HypSys contains -PPSPX- motifs, which have been identified as a repeating unit in hydroxyproline-rich glycoproteins, a major class of cell wall structural proteins (Narvaez-Vasquez et al. 2005; Pearce et al. 2001a). Interestingly, some SCOOPs, such as SCOOP2, also carry a -PPSPX- motif (Hou et al. 2021a). It is possible that these phytocytokines are associated with and released from cell walls, an interesting question to be explored in the future.

## Phytocytokines trigger overlapping and unique signaling pathways with MAMPs

**Fig. 2:**
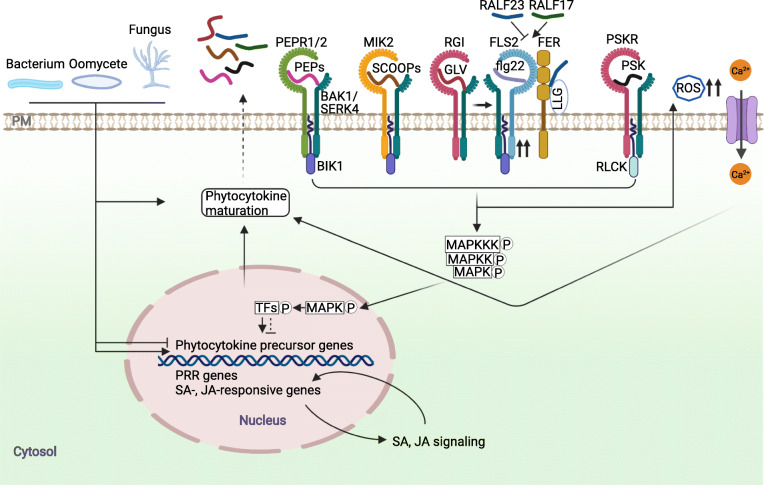
A proposed model of phytocytokine-mediated regulation of plant immunity. Pathogen infections swiftly activate or inhibit the expression of phytocytokine precursor genes, or promote phytocytokine maturation. When released to the apoplast, phytocytokines are perceived by their corresponding receptor- and co-receptors. These perceptions activate ROS burst, Ca^2+^ influx, and phosphorylation of MAP kinase kinase kinases (MAPKKKs) mediated by BIK1 and/or related RLCKs. Ca^2+^ may promote phytocytokine maturation. Activated MAPKs may phosphorylate transcription factors (TFs), which further up- or down-regulate the expression of phytocytokine precursor genes and PRR genes, and SA- and/or JA-responsive genes, thus amplifying or attenuating immunity. Phytocytokine signaling may also modulate PRR complex stability and assembly. For example, the complex formation between FLS2 and RGI induced by flg22 increases FLS2 abundance, and the association between FER, FLS2, and BAK1 is promoted or inhibited by RALF17 or RALF23, thus positively or negatively regulates PTI, respectively. Due to limited space, only several of well-studied phytocytokines and their receptors are shown in this Figure

Upon perception by cognate PRRs, MAMPs trigger convergent PTI responses, including phosphorylation of the receptor-like cytoplasmic kinases (RLCKs), the elevation of cytosolic Ca^2+^ concentration, transient apoplastic ROS burst, the activation of mitogen-activated protein kinases (MAPKs) and calcium-dependent protein kinases (CDPKs), reprogramming of defense gene expression, callose deposition, production of immune-related hormones and antimicrobial components, and plant growth inhibition (DeFalco and Zipfel, 2021; Yu et al. 2017; Zhou and Zhang 2020). Like MAMPs, some phytocytokines also activate canonical PTI responses (Fig. 2). For example, Pep1, PIP1, and SCOOP12 all trigger MAPK activation, ROS production, callose deposition, and induce the expression of some PTI marker genes (Gully et al. 2019; Hou et al. 2021a; Hou et al. 2014; Ranf et al. 2011; Rhodes et al. 2021). Since the expression of these phytocytokines and cognate receptors are induced by MAMPs, these phytocytokines were thought to amplify MAMP responses. In agreement with this, some flg22-induced responses or resistance to pathogens are partially compromised when these phytocytokine signaling pathways are disrupted (Gravino et al. 2017; Hou et al. 2014; Rhodes et al. 2021; Tintor et al. 2013). It is worth noting that SCOOP-MIK2 signaling promotes flg22- but antagonizes Pep1-induced ROS production (Rhodes et al. 2021), that complicates the crosstalk between MAMP- and phytocytokine-mediated immune signaling.

Moreover, the type and intensity of immune responses induced by phytocytokines could be different from that of MAMPs. The immune induction also differs between different phytocytokines, which may contribute to the diversification and specificity of plant immunity. For example, Pep1 and SCOOP12, like flg22, induce RLCK *BOTRYTIS-*INDUCED KINASE 1 (BIK1) phosphorylation and activate BIK1-depdendent immune responses, whereas PIP1 seems to induce immune responses through a BIK1-independent manner (Hou et al. 2021a; Hou et al. 2014; Liu et al. 2013; Tintor et al. 2013). In addition, Pep1 and SCOOP12 induce a strong immune response in roots but a weak response in leaves compared to flg22 (Hou et al. 2021a; Poncini et al. 2017). SCOOPs are able to cause root browning, a phenotype likely related to cell wall modifications and root immunity, which was not reported for flg22 or Pep1 (Felix et al. 1999; Hou et al. 2021a; Huffaker et al. 2006). Pep1 also activates distinct gene networks from flg22 in different cell types of roots (Rich-Griffin et al. 2020).

The plant defense hormones SA, ethylene (ET)/jasmonic acid (JA) function antagonistically in plants against biotrophic and necrotrophic pathogens, and all three hormones play an intertwined role in regulating flg22/elf18-triggered PTI (Kim et al. 2014). The specificity of phytocytokine-regulated immune signaling is also correlated with these immune-related hormones. For example, PIP1 and ZIP1 have been shown to activate SA signaling pathway and contribute to biotrophic pathogens (Hou et al. 2019b; Ziemann et al. 2018). In contrast, PSK, PSY1, and RALF activate JA signaling pathway to enhance plant resistance to necrotrophic pathogens and/or compromise plant resistance to hemibiotrophic pathogens (Guo et al. 2018; Mosher et al. 2013). Interestingly, Pep1 is able to activate both SA and ET/JA signaling pathways and promote plant resistance to both biotrophic and necrotrophic pathogens (Liu et al. 2013; Ross et al. 2014; Tintor et al. 2013; Yamaguchi et al. 2010). In addition, some phytocytokines also regulate plant immunity by crosstalking with some other hormone signaling pathways. For examples, PSK initiates auxin-dependent resistance to *B. cinerea* in tomato (Zhang et al. 2018), RALF suppresses ABA signaling although the role of this suppression in plant immunity remains to be explored (Chen et al. 2016; Yu et al. 2012).

## Phytocytokine signaling modulates PRR complex stability and assembly

The plasma membrane-resident receptors of both phytocytokines and MAMPs and their shared BAK1/SERK4 coreceptors provide a platform for the intertwined regulations between phytocytokine and MAMP receptors (Fig. 2). Phytocytokines and cognate receptors are able to modulate MAMP receptor complex assembly and signaling. FER forms a complex with EFR/FLS2 and BAK1, and promotes elf18/flg22-induced complex assembly between EFR/FLS2 and BAK1 in such strengthening PTI signaling (Stegmann et al. 2017). RALF23 perception by FER suppresses the ligand-induced EFR/FLS2-BAK1 complex formation (Stegmann et al. 2017). RALF23 may negatively regulate FER function in salt tolerance by inducing its internalization (Zhao et al. 2018), raising a possibility that RALF23 dampens the PTI signaling likely through a similar mechanism. Interestingly, RALF17, distinct from REALF23, promotes elf18-triggered ROS production via FER (Stegmann et al. 2017). It is possible that RALF17 strengthens the FER-mediated complex assembly between EFR and BAK1 by competing with RALF23 for FER binding. However, the mechanism underlying the RALF23 and RALF17 opposite modulation of the PRR complex-mediated signaling remains unclear. Additionally, FER closest homologs, ANXUR1 (ANX1) and ANX2 also associate with FLS2 and BAK1 (Mang et al. 2017). Likewise, flg22 perception by FLS2 promotes ANX1 association with BAK1. However, ANX1 negatively regulates flg22-induced FLS2-BAK1 complex formation and plant PTI responses (Mang et al. 2017). It would be interesting to test in the future if FER and ANX1 compete with each other for their association with the FLS2-BAK1 complex to either enhance or dampen PTI responses and if this FER-ANX balance is under the control of different RALF peptides. RGI3 forms an flg22-induced complex with FLS2, suggesting RGI3 is part of the activated PRR signaling platform (Stegmann et al. 2021). Interestingly, RGF9/GLV2 perception by RGI receptors increases FLS2 abundance and thus promotes FLS2 signaling output (Fig. 2) (Stegmann et al. 2021). It will be interesting to investigate the potential relationship between FLS2 abundance and RGI3-FLS2 association. FLS2 abundance is regulated by two E3 ligases plant U-box protein 12 (PUB12) and PUB13-mediated ubiquitination (Lu et al. 2011), raising the possibility that RGI3 interferes PUB12/PUB13-mediated ubiquitination of FLS2.

## Modulation of plant immunity by pathogen-encoded phytocytokine mimicry

Although phytocytokines are considered as plant-specific signaling molecules, some phytocytokine homologs or phytocytokine-like sequences are found in microbes, especially in plant pathogenic fungi, bacteria, and parasitic nematodes. Like phytocytokines in plants, most of microbial phytocytokine-like sequences are derived from C-termini of precursor proteins with an N-terminal signal peptide. These pathogen-encoded phytocytokine-like sequences are recognized by the same receptors and activate similar pathways with the corresponding phytocytokines in plants, and they are considered as phytocytokine mimics (Ronald and Joe 2018). The microbial phytocytokine mimics usually function as virulence factors to promote pathogen pathogenicity by hijacking phytocytokine-mediated cellular processes. However, some of these phytocytokine-like motifs are recently found to be capable of activating plant immune responses and are presumed to be potential MAMPs (Hou et al. 2021a; Rhodes et al. 2021).

Rice *Xanthomonas* resistance 21 (XA21), an LRR-RLK-type PRR, recognizes a synthetic 21-aa peptide with a sulfated tyrosine residue derived from the C-terminus of RaxX (RaxX21-sY) from the bacterial pathogen *X. oryzae* pv. *oryzae* (*Xoo*) strain PXO99, and activates immune responses for a broad-spectrum resistance to *Xoo* (Pruitt et al. 2015; Song et al. 1995). *Xanthomonas* strain lacking raxX is impaired in its ability to infect rice lacking XA21, suggesting that RaxX is a virulence factor. RaxX is a small protein with a predicted N-terminal signal peptide, which is highly conserved in many *Xanthomonas* species (Pruitt et al. 2015). Sequence analysis indicated that RaxX21 is similar to the phytocytokine PSY1 (Amano et al. 2007; Pruitt et al. 2017; Pruitt et al. 2015). Like PSY1, RaxX21-sY peptides from diverse *Xanthomonas* species promote plant root elongation, suggesting that RaxX21-sY is a functional mimic of plant PSYs. It was hypothesized that RaxX21-sY targets the plant cell surface-localized receptor of PSYs to elevate plant susceptibility to *Xanthomonas* strains*.* Although, PSY1R has been suggested to be a potential receptor of PSY1 in Arabidopsis (Amano et al. 2007), *psy1r* mutants are still responsive to both PSYs and RaxX21-sY (Pruitt et al. 2017), suggesting that an additional receptor(s) may be involved. Unlike RaxX21-sY, PSY peptides do not activate XA21-mediated immunity (Pruitt et al. 2017). This suggests that XA21 is a lately evolved receptor to specifically recognize RaxX21-sY and trigger plant resistance to RaxX-harboring pathogens.

Sequence alignments indicate that RALF homologs are not only widely distributed in plants but also across phylogenetically distant phytopathogens, such as pathogenic *Fusarium* fungi and Actinobacteria (Masachis et al. 2016; Thynne et al. 2017; Wood et al. 2020). The RALF-like proteins are also present in multiple species of root-knot nematodes (Masachis et al. 2016; Thynne et al. 2017; Zhang et al. 2020b). These microbial RALF homologs exhibit highly similar sequence characteristics with plant RALFs, including an N-terminal signal peptide and four highly conserved cysteine residues (Masachis et al. 2016; Thynne et al. 2017; Zhang et al. 2020b). Some pathogen RALF-like peptides have been shown to mimic plant RALFs and modulate FER-mediated responses, favoring the infection process of pathogens. For instance, the root-infecting fungus *F. oxysporum* secretes a functional RALF mimic (F-RALF) to induce extracellular alkalization by directly targeting FER to favor the fungal multiplication (Masachis et al. 2016). Likewise, the root-knot nematode *Meloidogyne incognita* contains two RALF-like genes (MiRALF1 and MiRALF3), which mimic host RALFs to bind FER, thereby manipulating FER-mediated signaling to promote *M. incognita* parasitism (Zhang et al. 2020b). Therefore, FER represents a virulence target of these microbial RALF-like factors. Two FER homologous CrRLK1L LETUM1 (LET1) and LET2 were recently reported to activate the nucleotide-binding domain leucine-rich repeat (NLR)-type immune receptor suppressor of *mkk1 mkk2* 2 (SUMM2)-mediated autoimmunity and cell death (Huang et al. 2020; Liu et al. 2020a). RALFs or related molecules could be the potential ligands of LET1/LET2 in regulating SUMM2 activation. It is tempting to speculate that RALFs or other phytocytokine mimics may function as “avirulence” factors to activate NLR-mediated immunity.

Although most RALF-like sequences identified in pathogens are close to Arabidopsis RALF1, pathogen-encoded RALF mimics intermix with plant RALFs without an apparent evolutionary origin with the phylogenetic analyses (Masachis et al. 2016; Thynne et al. 2017; Zhang et al. 2020b). Notably, *RALF-like* sequences found in the genomes of the poplar pathogen *Sphaerulina musiva* and *Septoria populicola* are close related to a poplar *RALF* gene (Thynne et al. 2017). These observations implicate pathogens may have acquired *RALF* genes by horizontal gene transfer from their host plants.

As an unidentified proteinaceous elicitor(s) isolated from *Fusarium* strains activates MIK2-mediated PTI responses, a functional analog(s) of SCOOPs is predicted to be encoded by *Fusarium* genomes (Coleman et al. 2021). Blast-searching with Arabidopsis SCOOPs revealed that some SCOOP-like (SCOOPL) motifs exist in different families of proteins in *Fusarium* strains (Hou et al. 2021a; Rhodes et al. 2021). However, distinct from classical phytocytokine mimics, all these SCOOPLs seem to be distributed in proteins belonging to different families. For example, one of the *Fusarium* SCOOPLs localizes in the N-terminus of a putative transcription regulator conserved in *Fusarium* strains (Hou et al. 2021a); another *Fusarium* SCOOPL is present in the C-terminus of a DNA topoisomerase (Rhodes et al. 2021). In addition, SCOOPLs also exist in an unknown function protein conserved in bacterial *Comamonadaceae* (Hou et al. 2021a). Importantly, synthetic peptides corresponding to some of these SCOOPLs in *Fusarium* and *Comamonadaceae* are functional in activating MIK2- and/or BAK1/SERK4-dependent immune responses, though the activities are weaker than Arabidopsis SCOOPs (Hou et al. 2021a; Rhodes et al. 2021). Knock-out of a *SCOOPL* in *F*. *oxysporum* 5176 enhanced the fungal pathogenicity in Arabidopsis (Hou et al. 2021a). Therefore, SCOOPLs may function as MAMPs, rather than virulence factors, to activate MIK2-BAK1/SERK4-mediated PTI responses.

Compared to the wide distribution of SCOOPLs in fungal *Fusarium* spp. and bacterial *Comamonadaceae*, plant SCOOPs are only present in *Brassicaceae* plants and are undergoing a significant gene expansion (Gully et al. 2019). This suggests that plant *SCOOPs* may have evolved later than microbial *SCOOPL*s. In addition, phylogenetic analysis indicates that peptide motifs of Arabidopsis SCOOPs, *Fusarium* and *Comamonadaceae* SCOOPLs might have evolved independently (Hou et al. 2021a). Moreover, plant SCOOPs are derived from small peptide precursor proteins, whereas *Fusarium* and *Comamonadaceae* SCOOPLs reside in proteins belonging to distinct families. The divergence of protein families harboring SCOOP/SCOOPL also suggests that SCOOPs and SCOOPLs might have evolved convergently but unlikely by horizontal gene transfers. Thus, it’s predicted that plant SCOOPs may have been convergently evolved to mimic microbial SCOOPLs and amplify SCOOPL-triggered immunity (Hou et al. 2021a).

## Concluding remarks and perspectives

Plant endogenous peptide signaling has been discovered to be involved in the regulation of plant immunity for a long time. These immunomodulatory peptides were recently defined as “phytocytokines”, a term derived from “cytokines” as a group of peptides functioning in metazoan immune system. Recent progresses have uncovered that phytocytokines, like cytokines, are produced and released into extracellular compartments when plants experience pathogen invasions; phytocytokines, like MAMPs and DAMPs, are recognized by plasma membrane-localized receptors to activate canonical PTI responses or regulate plant immunity through a unique signaling mechanism. However, more phytocytokines and their cognate receptors still await to be identified in plants especially in crops. The functional specificity and coordination between different phytocytokines of intra- and interfamily remains largely unknown. Future efforts are needed to decipher how phytocytokines specialize plant resistance to a class of pathogens and how different phytocytokines coordinate to achieve a broad-spectrum of plant resistance.

Several phytocytokine-like sequences have been identified in microbes. These microbe-encoded phytocytokine-likes seem to function as virulence factors or MAMPs to dampen or activate plant immunity. Besides, many phytocytokines are perceived by receptors evolutionarily closed to the receptors of some proteinaceous MAMPs. It implies an evolutionary relevance between phytocytokine signaling and MAMP signaling. A systematic and comparative analysis of phytocytokine-likes in microbes at the genome level may shed new light on the evolution of plant immunity. PTI and effector-triggered immunity (ETI), two tiers of plant immune pathways, have been recently shown to potentiate each other (Ngou et al. 2021; Yuan et al. 2021). It will be interesting to determine the potential involvement of phytocytokines in plant ETI, and whether phytocytokine signaling could function as a connecting point between plant PTI and ETI. Finally, some immunological phytocytokines also function in developmental processes and plant tolerance to diverse abiotic stresses. The molecular mechanisms underlying signaling crosstalks between phytocytokine-mediated different physiological processes need to be investigated. Addressing these questions will advance our understanding of the phytocytokine functions and elucidate how plants integrate different stress responses through phytocytokine signaling.

## Data Availability

Not applicable.

## Abbreviations

ANX1: ANXUR1
BAK1: BRASSINOSTEROID INSENSITIVE 1 (BRI1)-ASSOCIATED RECEPTOR KINASE 1
BIK1: *BOTRYTIS*-INDUCED KINASE 1
CDPKs: Calcium-dependent protein kinases
CrRLK1L: *C. roseus* receptor-like kinase 1-like
DAMPs: Damage-associated molecular patterns
ETI: Effector-triggered immunity
EFR: ELONGATION FACTOR-Tu RECEPTOR
EF-Tu: Elongation factor Tu
ER: Endoplasmic reticulum
ET: Ethylene
FER: FERONIA
FLS2: FLAGELLIN SENSING 2
IDA: Inflorescence deficient in abscission
IDL6: IDA-LIKE 6
GLVs: GOLVENs
HSL2: HAESA-LIKE 2
HypSys: Hydroxyproline-rich systemins
JA: Jasmonic acid
LET1: LETUM1
LLG1: LORELEI (LRE)-LIKE GLYCOSYLPHOSPHATIDYLINOSITOL (GPI)-ANCHORED PROTEIN 1
MAPKs: Mitogen-activated protein kinases
MC4: METACASPASE 4
MIK2: MALE DISCOVERER 1-INTERACTING RECEPTOR-LIKE KINASE 2
PAMPs/MAMPs: Pathogen/microbe-associated molecular patterns
PEP1: Plant elicitor peptide 1
PEPR1: PEP1 RECEPTOR 1
PIP1: PAMP-induced secreted peptide 1
PLCP: Papain-like cysteine protease
PM: Plasma membrane
PRRs: Pattern recognition receptors
PS: Prosystemin
PSKs: Phytosulfokines
PSKR1: PSK RECEPTOR 1
PSY1: Plant peptide containing sulphated tyrosine 1
PTI: Pattern-triggered immunity
RALFs: Rapid alkalization factors
RGFs: Root meristem growth factors
RGI3: RGF1 INSENSITIVE 3
RLCKs: Receptor-like cytoplasmic kinases
RLKs: Receptor-like kinases
RLK7: RECEPTOR-LIKE 7
SA: Salicylic acid
SCOOP12: Serine-rich endogenous peptide 12
SCOOPL: SCOOP-like
SERK: SOMATIC EMBRYOGENESIS RECEPTOR-LIKE KINASE
*Sl*Phyt1: SLPHYTASPASE 1
SUMM2: Suppressor of *mkk1 mkk2* 2
SYR1: SYSTEMIN RECEPTOR 1
S1P: SITE-1 PROTEASE
XA21: *Xanthomonas* resistance 21
ZIP1: *Z. mays* immune signaling peptide
